# LDL receptor knock-out mice show impaired spatial cognition with hippocampal vulnerability to apoptosis and deficits in synapses

**DOI:** 10.1186/1476-511X-13-175

**Published:** 2014-11-20

**Authors:** Shao-hua Wang, Yan Huang, Yang Yuan, Wen-qing Xia, Pin Wang, Rong Huang

**Affiliations:** Department of Endocrinology, ZhongDa Hospital of Southeast University, No.87 DingJiaQiao Road, Nanjing, 210009 PR China; Department of Endocrinology, YanCheng First People’s Hospital, No.16 YueHe Road, YanCheng, 224005 PR China

**Keywords:** LDL receptor knock-out, Cognition, Synapse, Apoptosis

## Abstract

**Background:**

Evidence from clinical studies support the fact that abnormal cholesterol metabolism in the brain leads to progressive cognitive dysfunction. The low-density lipoprotein receptor (LDLR) is well-known for its role in regulating cholesterol metabolism. Whether LDLR involved in this impaired cognition and the potential mechanisms that underlie this impairment are unknown.

**Methods:**

Twelve-month-old Ldlr-/- mice (n = 10) and wild-type littermates C57BL/6 J (n = 14) were subjected to the Morris water maze test. At 1 week after completion of the behavioural testing, all of the animals were sacrificed for analysis of synaptic and apoptotic markers.

**Results:**

The plasma cholesterol concentration of Ldlr-/- mice was increased moderately when compared with C57BL/6 J mice (*P* < 0.05). Behavioural testing revealed that Ldlr-/- mice displayed impaired spatial memory, and moreover, the expression levels of synaptophysin and the number of synaptophysin-immunoreactive presynaptic boutons in the hippocampal CA1 and dentate gyrus were decreased (all *P* < 0.05). Ultrastructural changes in the dentate gyrus were observed using transmission electron microscopy. Furthermore, apoptosis in the hippocampus of Ldlr-/- mice was revealed based on elevation, at both the mRNA and protein levels, of the ratio of Bax/Bcl-2 expression (all *P* < 0.05)and an increase in activated-caspase3 protein level (*P* < 0.05).

**Conclusion:**

LDLR deficiency contributes to impaired spatial cognition. This most likely occurs via negative effects that promote apoptosis and synaptic deficits in the hippocampus.

## Introduction

Alzheimer’s disease (AD) is characterised by extracellular amyloid plaques, intra-neuronal neurofibrillary tangles, cerebrovascular amyloid deposits and a loss of neurons and synapses in specific brain regions [1, 2]. The mechanisms are not fully understood. Accumulating evidence from clinical [3–5], epidemiological [6–8], animal, cell culture [9] and genetic studies [2, 8, 10–12] suggests links between brain cholesterol and the development of AD. Disturbed cerebral cholesterol metabolism is now gaining attentions as a crucial aetiological factor underlying AD. Cholesterol metabolism in the brain is independent of levels in plasma [13]. Cerebral cholesterol is highly enriched and is synthesised de novo by glial cells, which also synthesise apoE. The cholesterol is transported by apoE and is taken up by neurons via specific receptors, primarily the LDLR, for synaptogenesis and axonal growth [14].

In the periphery, the LDLR is well-known for its important role in removing cholesterol-rich lipoprotein particles from the plasma to maintain cholesterol and apoE homeostasis. In humans, LDLR deficiency causes familial hypercholesterolaemia [15, 16]. LDLR is widely expressed in the brain [17–21] and as an important receptor for apoE, LDLR plays a key role in brain cholesterol metabolism and brain function. A cohort study has revealed that LDLR-deficient patients showed a high incidence of mild cognitive impairment [22]. Genetic studies have revealed that LDLR is one of five cholesterol-related genes that show the most promising association with a risk of developing AD [3, 23]. In AD mouse models, LDLR deficiency aggravates learning deficits and leads to increased apoE levels and amyloid deposits [24, 25]. Moreover, the overexpression of LDLR in the brain decreases apoE levels and inhibits amyloid deposition markedly [26]. Furthermore, Ldlr^-/-^ mice show modified transbilayer distribution of cholesterol in synaptic plasma membrane preparations from the brain [27].

The hippocampus has frequently been used as a model system of the brain and used widely for neurological studies. The hippocampus is the key region underlying learning and memory formation, and its normal function depends upon the integrity of its neurons and synapses. The LDLR is widely distributed in the hippocampus [18]. LDLR plays a key role in cholesterol metabolism in the brain. In regard to the hippocampus, we hypothesised that an absence of LDLR may impair hippocampal function and negatively impact cognitive function. In this study, using Ldlr^-/-^ mice, we imitated disordered cholesterol metabolism in the brain and examined its influence on cognitive performance and the possible underlying mechanisms. Our results will be helpful in understanding the role of LDLR in cholesterol-related cognitive impairment.

## Experimental procedures

### Animals and experimental design

A total of 24 four-month-old male LDLR deficient (Ldlr^-/-^) mice (n = 10) and C57BL/6 J littermates (n = 14) were purchased from the Model Animal Resource Information Platform of Nanjing University. The Ldlr^-/-^ mice (stock #002207, strain name B6.12957-Ldlr^tm1Her^) were introduced from the Jackson Laboratory (Bar Harbor, ME) in 2002 and were originally generated by homologous recombination in cultured embryonic stem cells to produce mice that lack functional LDLR. The mice were then backcrossed to C57BL/6 J mice for ten generations to obtain homozygous Ldlr^-/-^ mice on a C57BL/6 J background [28]. The mice were housed under standard conditions in conventional cages and had free access to food (standard rodent chow diet, Xietong Organism, Jiangsu, China) and water and were maintained at 21 ± 2°C on a 12 h light/dark cycle (lights on at 7:00 a.m.). All of the experiments were conducted in compliance with the National Institute of Health Guidelines for the Care and Use of Laboratory Animals (NIH Publications No.80-23), revised in 1996.The Animal Care and Use Committee of the Southeast University approved this animal study.

After one week of environmental adaptation, both the Ldlr^-/-^ and WT C57BL/6 J mice were subjected to a Morris water maze test to determine their baseline cognitive performance, and the test was performed again 8 months later when the mice were 12 months old. The testing was conducted and scored by experienced observers who were blind to the genetic background of the mice. The body weight of each mouse was measured monthly. At 1 week after the completion of the final behavioural test, the plasma lipids of mice were examined as described previously [29]. The mice were anesthetised using intraperitoneal sodium pentobarbital (80 mg/kg) and killed. To perform the varied methods that are used for the study of apoptotic and synaptic markers, the hippocampal tissue was extracted immediately before or after mice were perfused transcardially via a left ventricular cannula with 4% phosphate-buffered paraformaldehyde (pH 7.4). At least three mice from each group were used for each measurement.

### Morris water maze test

To evaluate the spatial learning and memory of the mice, Morris water maze testing was used as described previously [30, 31]. The mice were tested between 8:00 am and 12:00 pm. The water maze pool (diameter, 122 cm;height, 50 cm) was filled to a depth of 30 cm using water (25 ± 2°C) made opaque using milk powder. A black platform (8 cm diameter, 1 cm below the surface of the water) was hidden and was used by the mice as the escape from swimming. The test included spatial acquisition and a probe test and was performed over 5 days. For the spatial acquisition test, during the first four days, the mice were trained with 3 trials per day to locate the platform. The interval between the 3 trials per day is 30 minutes. They were given a maximum of 60 sec to locate the hidden platform and were permitted a stay of 10 sec on the platform. Those who failed to locate the platform were guided to it by the experimenter and were also permitted a stay of 10 sec. For analysis, the pool was subdivided into 4 equal quadrants labelled N, S, E and W. The time required to reach the platform (escape latency), the distance swam to the platform, swimming speed and the percentage of time spent swimming in the target quadrant were recorded using a video tracking system (EthoVision Image Analysis 3.1, Noldus Information Technology, Wageningen, The Netherlands). A probe test to assess the degree of memory consolidation after learning was conducted on the fifth day by removing the platform. Each mouse was given a single 60 sec trial, and the time taken to reach the original position of the platform and spent in the target quadrant and the number of platform crossovers was recorded.

### Electron microscopy

Damage to the DG region of the hippocampus often prevents the growth of new born cells during the critical period of memory formation, so ultrastructural changes in the DG were observed using transmission electron microscopy. The DG tissue was extracted under a dissection microscope (Zeiss, Germany) and 1-mm^3^ tissue blocks were generated that were fixed and subsequently treated as described previously [32, 33]. Morphological changes in nerve cells and synapses were observed using an AMTXR60 digital camera attached to a JEM 1400 transmission electron microscope. We identified mitochondria by the presence of distinctive cristae and a double membrane and mature synapses by the presence of the following features on at least one section: a postsynaptic density, at least three synaptic vesicles within 100 nm of the presynaptic membrane and a clearly defined synaptic cleft.

### Synaptophysin detection

With regard to synaptic density, we evaluated the number of presynaptic boutons using synaptophysin immunostaining and the gene and protein expression levels of synaptophysin. For synaptophysin immunostaining, the hippocampus was cut into a series of 25-μm thick coronal sections using a cryostat (Leica CM 3050; Leica, Nussloch, Germany). For each animal, one subseries of 7 (every 10th) sections was used for free floating immunofluorescence processing and synaptic density analyses. The sections were incubated in PBS-T(0.3%Triton X-100 in PBS, pH7.4) for 1 h, then in blocking solution for 2 h, and then with rabbit monoclonal anti-synaptophysin(Abcam, 1:1000) overnight at 4°C. After washing, the sections were incubated for 2 h with FITC-conjugated goat anti-rabbit IgG (Jackson, 1:100) and then incubated for 10 min with DAPI to stain the nuclei. To quantify the density of synaptophysin-immunoreactive presynaptic boutons (SIPBs), CA1 and DG regions within the hippocampus of both hemispheres were assessed in each section. Three randomly chosen areas of the CA1 and DG were examined by capturing confocal images using a digital camera (F-view; Olympus, Tokyo, Japan) attached to an Olympus AX-70 microscope (100× oil immersion objective and a 10× projection lens). In total, 12 confocal images per section and 84 images per animal were obtained (3 images × 2 areas × 2 hemispheres × 7 sections). For the quantification of the synaptophysin fluorescence signal, images of labelled boutons were acquired using the same settings. SIPBs were measured using Image-Pro Plus software (Media-Cybernetics, Silver Spring, MD), as previously described [34, 35]. Appropriate background correction was first completed, and the immunoreactive presynaptic boutons were then counted in sampling fields. The SIPB density per unit area (100 μm^2^) was calculated. The data from individual animals of each group were pooled.

### Real-time RT-PCR

Real-time RT-PCR was used to investigate the mRNA expression level of synaptophysin and the apoptosis-related genes bcl-2, bax and caspase3 to analyse synaptic density and neuronal apoptosis. Total RNA was extracted from the hippocampus using a trizol reagent kit (Invitrogen). Total RNA (1 μg) was used as a template for first-strand cDNA synthesis using random primers and the Promega RT System. All of the PCR primers were designed using the Primer Premier 5.0 software (Table 1). The cDNA template (1 μL) was mixed into the Master Mix (including 10 × SYBR Green PCR buffer, 25 mM MgCl_2_, 2.5 mM dNTP, 10,000× SYBR Green and Taq DNA polymerase) together with 20 μM of each primer. The reaction mixture was brought up to a final volume of 25 μL with RNase-free deionised water. The amplification conditions were 2 min at 30°C, 10 min at 95°C (Taq DNA polymerase activation), followed by 40 cycles of 20 s at 94°C (denaturing), 20 s at 55°C (annealing) and 30 s at 72°C (extension). Real-time RT-PCR was performed by monitoring the increase in fluorescence intensity of the SYBR Green dye using a Rotor-Gene 3000 real-time PCR apparatus (Corbett Research) according to the manufacturer’s instructions. All of the measurements were performed in triplicate. The real-time RT-PCR data are represented as Ct values, where the Ct was defined as the threshold PCR cycle where the amplified product was first detected. Tominimise intra- and inter-assay variability caused by differences in PCR efficiency, 5 replicates were performed. The Ct or threshold value of the target sequence is directly proportional to the absolute concentration when compared with the threshold value of the reference genes. The relative expression levels of bcl-2, bax, caspase3 and synaptophysin were plotted as the fold change compared with the control and were determined using the 2^-ΔΔCt^method (21). This method consists of analgorithm that calculates the relative concentration. The factor X, which is the fold change in the expression of the gene, can be calculated using the following formula: X = 2^-ΔΔCt^ where ΔΔCt = (Ct of the target gene – Ct of NAPDH) of the control group - (Ct of the target gene - Ct of NAPDH) of the sample group.

**Table 1:** **The primers for real-time RT-PCR**

Genes	Primers
Bcl-2	5′- CTGGTGGACAACATCGCTCTG-3′ sense
	5′- GGTCTGCTGACCTCACTTGTG-3′ antisense
Bax	5′- TCCAGGATCGAGCAGA-3′ sense
	5′- AAGTAGAAGAGGGCAACC-3′ antisense
Caspase-3	5′- CATGGCCTGTCAGAAAATAC-3′ sense
Synaptophysin	5′- TAACCCGAGTAAGAATGTGC-3′ antisense
	5′- CCCTACATTCACCCACTTCTCC-3′ sense
	5′- TTATCTCCTCTCTGCCCGTTTC-3′ sense
GAPDH	5′- TGTTCCTACCCCCAATGTGTCCGTC-3′ sense
	5′- CTGGTCCTCAGTGTAGCCCAAGATG-3′ antisense

### Western blotting

The samples of hippocampal tissue were dissected on ice and washed three times using cold PBS. They were then homogenised in ice-cold lysis buffer (20 mM Tris, pH 7.5, 150 mM NaCl, 1 mM EDTA, 1 mM EGTA, 1%TritonX-100, 2.5 mM Na-pyrophosphate, 1 mM β-glycerophosphate, 1 mM Na_3_VO_4_, 1 g/mL leupeptin and 1 mM PMSF). The samples were centrifuged at 14,000 rpm for 15 min. The protein concentration of the supernatant was determined using a spectrophotometer (Thermo). The loading buffer was added, and the samples were boiled for 3 min. Protein (50 μg) was then loaded onto a 10% Bis-Tris gel (Invitrogen). The bands were transferred to apolyvinylidene fluoride (PVDF) membrane, which was then blocked using Tris-buffered saline (TBS) containing 1% bovine serum albumin and 5% non-fat milk powder (w/v). The membrane was then incubated overnight at 4°C with anti-bcl-2 (1:1000), anti-bax (1:1000), anti-activated-caspase3 (1:1000) and anti-synaptophysin (1:1000; all from Abcam) primary antibodies. Immunoreactivity was detected using horseradish peroxidase-conjugated secondary antibodies and visualised using enhanced chemiluminescence. The expression levels of each target protein and a GAPDH internal control were quantified using densitometry (TotalLab, version 1.1, UK), and the intensity of each target protein signal was normalised to GAPDH. All of the data were expressed relative to the control and represent the relative expression of each target protein.

### Statistical analyses

The data were analysed using SPSS16.0. The values are represented as the means ± standard deviations (S.D.) where indicated. A probability value of *P* < 0.05 was considered to be statistically significant. MWM spatial acquisition and body weights were analysed using a repeated measures analysis of variance (ANOVA) with genotype (control and Ldlr^-/-^) as the between-subjects factor and time (days 1–4 and months 1–8) as the within-subjects factor, respectively. Bonferroni-corrected *t*-tests were further used to examine significant differences between experimental time points or between animal groups. The MWM probe test, serum cholesterol, immunofluorescence staining, real time RT-PCR, western blotting and electron microscopy data were analysed using *t*-tests.

## Results and discussion

### Body weight gain and lipid analyses

**Figure 1:**
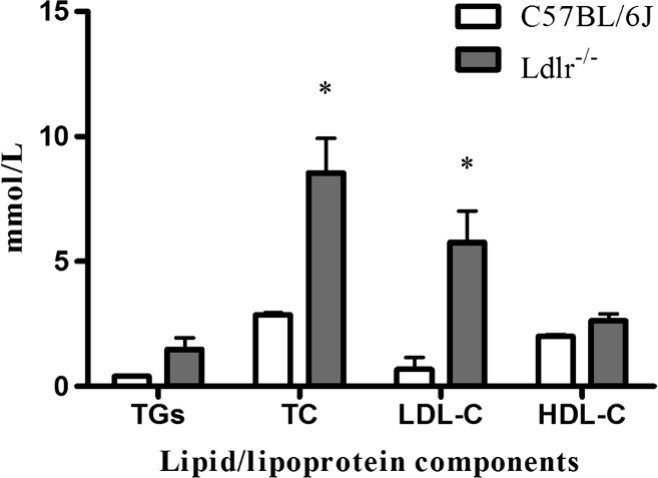
**Lipid analysis in C57BL/6 J and Ldlr** ^**-/-**^ **mice fed a standard rodent chow diet.** Total triglycerides (TGs), cholesterol (TC), LDL-c and HDL-c levels in plasma samples collected from 12-h-fasted animals after completion of behavioural testing. *P <0.05 for Ldlr^-/-^ vs. C57BL/6 J mice.

The Ldlr^-/-^ mice appeared healthy, and no differences in physical appearance or behaviour in the home cage was apparent between the groups. Repeated measures analysis of variance showed that the body weight of both groups increased steadily (*F*(8,196) = 197.83, *P* < 0.001) over the 8-month period. There was no statistically significant effect of Ldlr deficiency on body weight gain (*F*(1,22) = 0.15, *P* = 0.71). The weight of the C57BL/6 J and Ldlr^-/-^ mice was 42 ± 4 g and 42 ± 2 g by 12 months of age. With regard to plasma lipids, we determined the levels of plasma cholesterol, triglyceride, HDL-c and LDL-c. Total (fasting) plasma cholesterol (TC) values were approximately three-fold higher in the Ldlr^-/-^ mice compared with theC57BL/6 J mice (*t*(4) = 4.08, *P* = 0.02) due to an eight-fold increase in LDL-c (*t*(4) = 3.77, *P* = 0.02), whereas there was no significant change in HDL-c (*t*(4) = 2.31, *P* = 0.14). There was no significant difference in plasma levels of triglycerides between the two groups (*t*(4) = 2.13, *P* = 0.10) (Figure 1).

### Morris water maze test

During the baseline MWM test, the escape latencies (*F*(3,66) = 10.20, *P* < 0.001), distances (*F*(3,66) = 15.16, *P* < 0.001) and swim speeds (*F*(3,66) = 11.52, *P* < 0.001) decreased in both groups over the 4-day spatial acquisition test period indicating that all of the mice learned the task quickly. However, neither these three above-mentioned indices nor the target quadrant time percentage differed between the groups. For the probe trial test, no differences in the time taken to reach or to stay in the target square, the distance swam and the swim speed or platform crossovers were found between the two groups. These results indicated that Ldlr^-/-^ mice did not show impaired spatial cognition at baseline.

**Figure 2:**
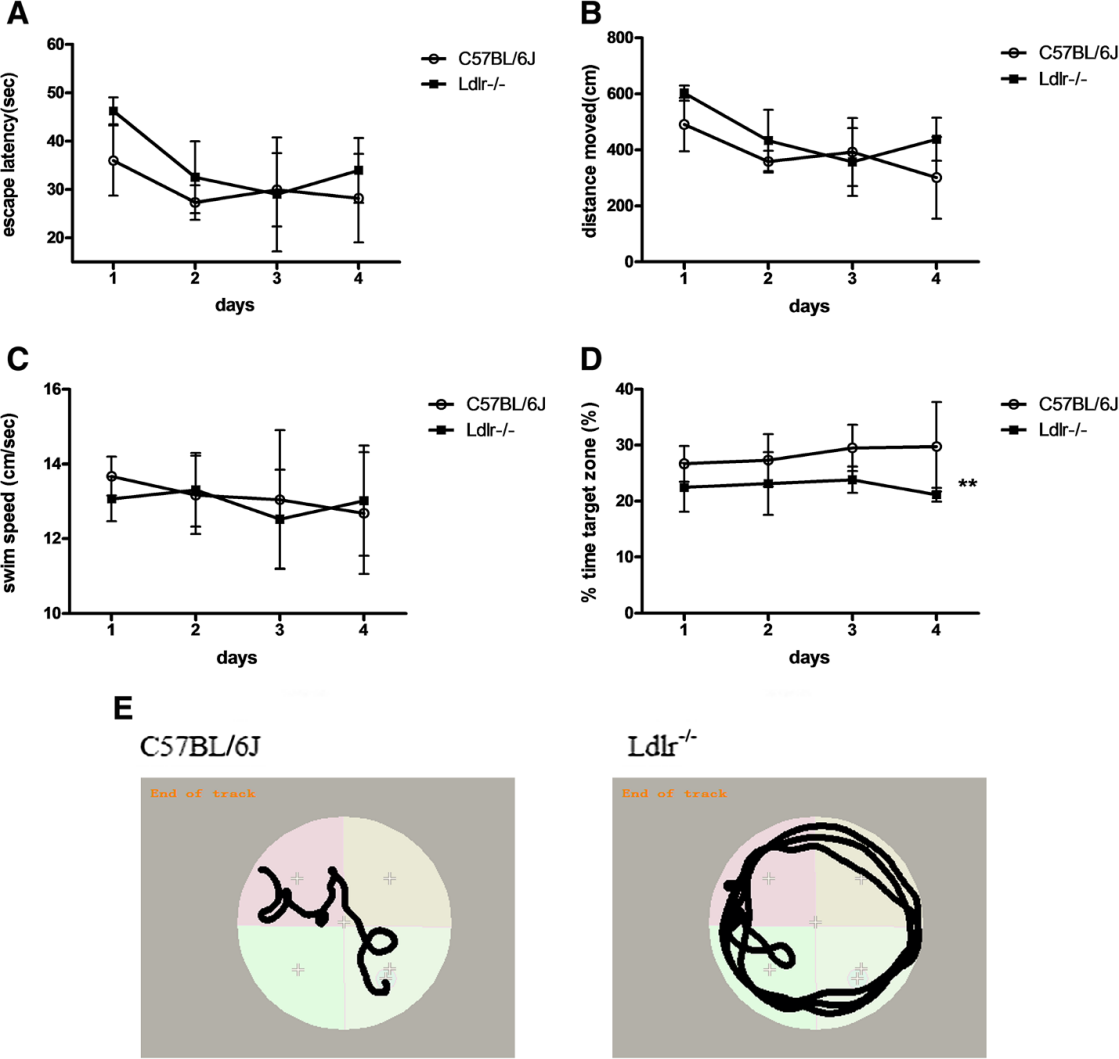
**The spatial acquisition phase of the MWM task.** The data shown are escape latencies **(A)**, distances moved **(B)**, swim speed **(C)**, the percentage of time spent in the target quadrant **(D)** and representative swim paths **(E)** during the spatial acquisition test (three trials per day over four successive days) of the MWM task in C57BL/6 J (n = 14) and Ldlr^-/-^ mice (n = 10) groups. **P < 0.01 for Ldlr^-/-^ vs. C57BL/6 J mice.

**Figure 3:**
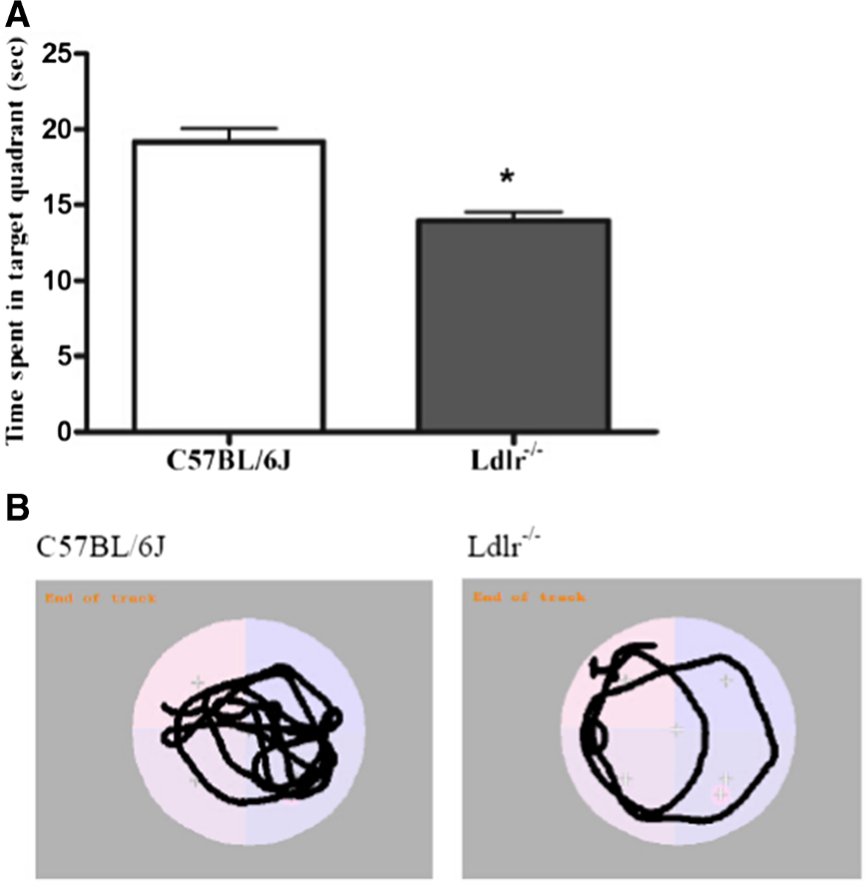
**The probe test of the MWM task.** The data shown are the time spent in the target quadrant **(A)** and representative swim paths **(B)** during the probe test (performed on the fifth day after the first four days of training). *P < 0.05 for Ldlr^-/-^ vs. C57BL/6 J mice.

Eight months later, the MWM test indicated that during the spatial acquisition test period, the escape latencies (Figure 2A) and distances swam (Figure 2B) to reach the hidden platform were similarly decreased in both groups over the four testing days (*F*(3, 66) = 8.68, *P* < 0.01). No significant differences in swim speed were noted between the two groups (Figure 2C). Repeated measures analysis of variance (ANOVA) showed significant effects of Ldlr deficiency on the reduced percentage of time spent swimming in the target quadrant (*F*(3,66) = 10.90, *P* = 0.01) (Figure 2D), showing a poorer learning performance. Subsequent Bonferroni post-hoc tests confirmed that the percentage time was significantly less on day 3 (*t* = 2.74, *P* = 0.02) and day 4 (*t* = 2.79, *P* = 0.03) in Ldlr^-/-^ mice. Typical swimming patterns observed on day 4 of the training period suggested that C57BL/6 J mice found the platform primarily by tendency and linear patterns, whereas the Ldlr^-/-^ mice swam at the margins, and the swimming patterns were random (Figure 2E). For the probe trial, the platform was removed, and the Ldlr^-/-^ group failed to remember the precise location of the platform and spent significantly less time in the target quadrant (*t* = 4.52, *P* = 0.001; Figure 3A). No statistically significant differences were noted between the two groups in the time spent reaching the target square,the distance swam or swim speed or in platform crossovers. Typical swimming patterns (Figure 3B) during the probe test showed that the C57BL/6 J mice looked for the platform by shuttling back and forth. In contrast to the C57BL/6 J mice, Ldlr^-/-^ mice swam along the pool border to search for the platform using patterns at the margin.

### Electron microscopy

**Figure 4:**
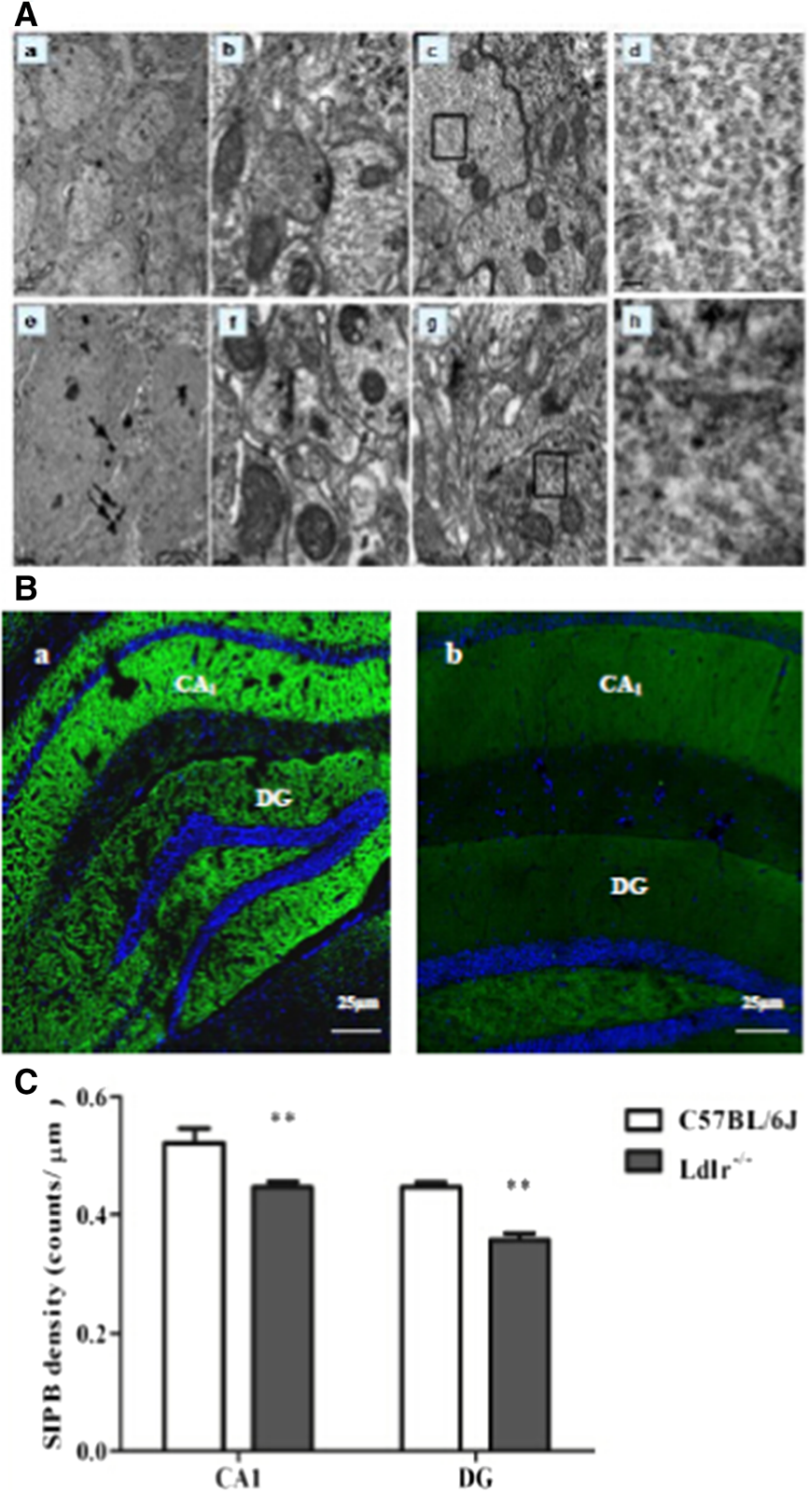
**Pathological changes in the hippocampus. A**: Electron microscopic examination of the DG stratum radiatum. The data of C57BL/6 J mice shown in a-d are ultrastructural analyses of DG general tissue (a), mitochondrial and synapse ultrastructure (“*” in b), neurofibrils (“□”in c) which is amplified in (d). the data of Ldlr-/- mice shown in e-h are glial cell lipid deposition (“→”in e), ultrastructural loose arrangement and oedema (“#”in f), a decrease in synaptic vesicles (“*” in f) , and sparse neurofibrils (“□”in g) which is amplified in (h). Scale bar = 2 μm in a and e; 0.2 μm in b, c, f and g; 0.04 μm in d and h. **B**: Density of synaptophysin-immunoreactive presynaptic boutons (SIPBs) in the hippocampus of Ldlr-/- mice. A representative image of the hippocampus of a C57BL/6 J mouse (a) and Ldlr-/- mouse (b) at 20 × magnification is shown. Quantitative data showing a significant decrease in SIPBs in both the CA1 and DG subregions in Ldlr-/- mice **(C)**. Scale bar = 25 μm in a and b. **P < 0.01 for Ldlr-/- vs. C57BL/6 J mice.

Ultrastructural analysis of the dentate gyrus revealed robust differences between Ldlr-/- and C57BL/6 J mice. In Ldlr-/- mice, lipid particles were widely distributed in the cytoplasm of glial cells indicating increased intracellular lipid deposition (the “→” label in Figure 4Ae). The ultrastructural arrangement was loose and showed oedema (the “#” label in Figure 4Af). The morphology of the neurofibrils was sparse, and their arrangement was disordered (the “□” label in Figure 4Ag) which is amplified in (Figure 4Ah). Analyses of synapses showed wider synaptic clefts between the two synaptic membranes and decreased synaptic vesicles (the “*” label in Figure 4Af). In tissue from C57BL/6 J mice, the mitochondria, neurofibrils and synapses showed no evidence of lipid deposition (Figure 4Aa). Neatly arranged mitochondrial cristae and neurofibrils as well as normal synaptic clefts and synaptic vesicles were also observed (Figure 4Ab-c).

### Synaptophysin detection

Synaptic density was measured in the hippocampal CA_1_ and DG regions using synaptophysin immunoreactivity. Representative images of synaptophysin-stained presynaptic boutons are shown in Figure 4Ba-b. As shown in Figure 4Bc, the SIPBs in the hippocampal CA_1_ and DG regions were lower in Ldlr^-/-^ mice than in C57BL/6 J mice (*t* = 3.50, *P* = 0.001; *t* = 6.38, *P* < 0.001, respectively).

**Figure 5:**
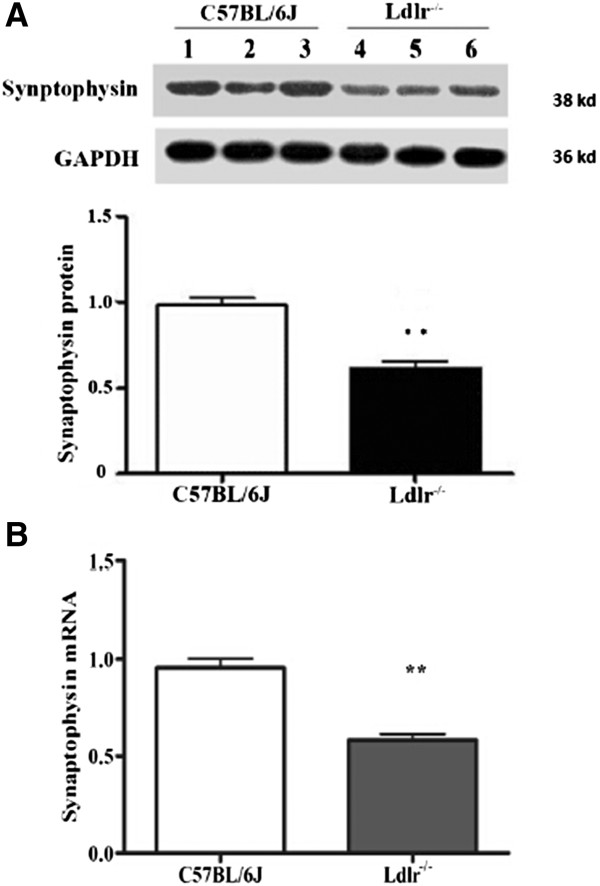
**Effects of LDLR knockout on synaptophysin expression in the hippocampus of C57BL/6 J and Ldlr** ^**-/-**^ **mice.** The data shown are of mRNA **(B)** and protein **(A)** levels of synaptophysin in the hippocampus. **P < 0.01 for Ldlr^-/-^ vs. C57BL/6 J mice.

The mean protein (Figure 5A) and mRNA (Figure 5B) expression levels of synaptophysin in the Ldlr^-/-^ mice, which showed cognitive decline, were lower than in the C57BL/6 J mice (*t* = -6.05, *P* = 0.004; *t* = -6.53, *P* = 0.003, respectively).

### Apoptosis and apoptosis-related genes

**Figure 6:**
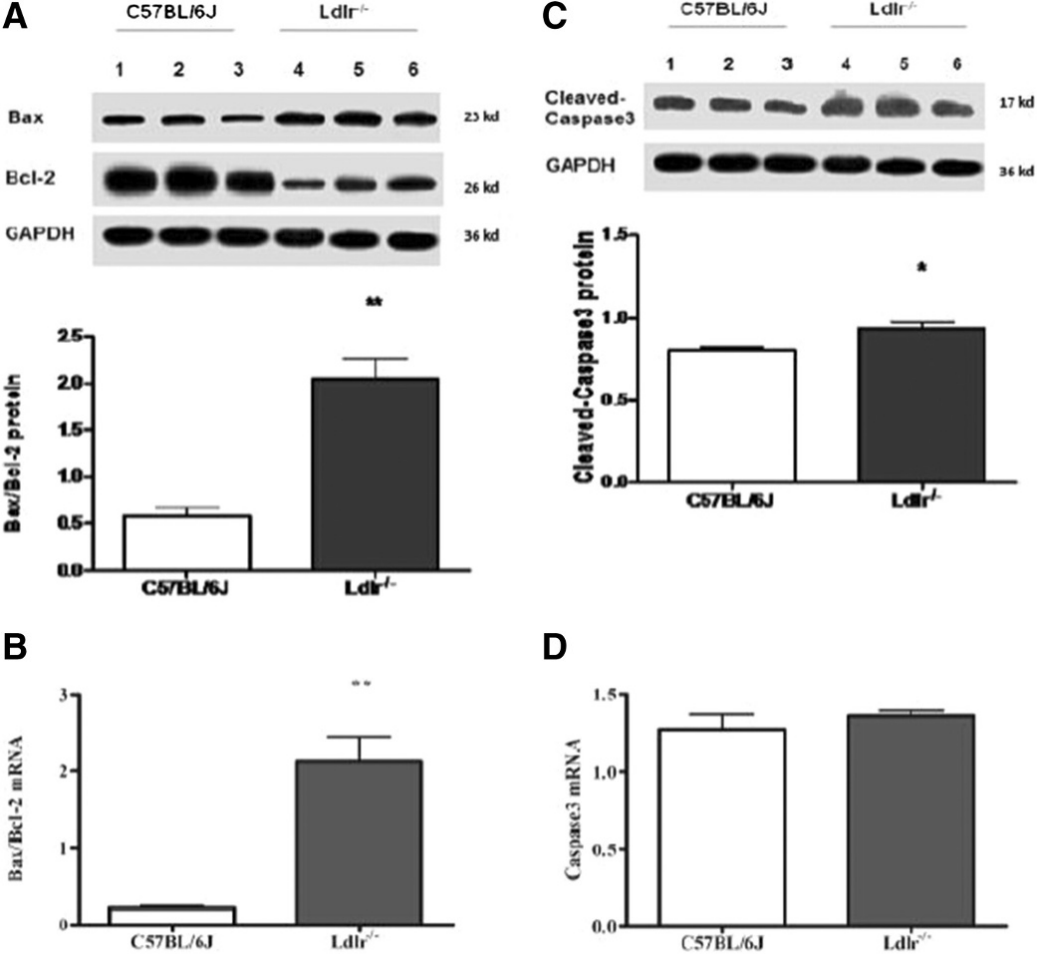
**Effects of LDLR knockout on expression of the apoptosis-related genes Bax, Bcl-2 and caspase3 in the hippocampus of C57BL/6 J and Ldlr** ^**-/-**^ **mice.** The data shown are the ratio of protein **(A)** and mRNA **(B)** levels of Bax to Bcl-2, the levels of activated-caspase3 protein **(C)** and caspase-3 mRNA **(D)** are also quantified in the hippocampus. *P < 0.05, **P < 0.01 for Ldlr^-/-^ vs. C57BL/6 J mice.

The mean ratio of Bax/Bcl-2 protein (Figure 6A) and mRNA (Figure 6B) was approximately 5-6-fold higher in Ldlr^-/-^ mice compared with C57BL/6 J mice (*t* = 6.16, *P* = 0.004; *t* = 4.37, *P* = 0.01, respectively). Individually, the mean levels of Bax protein and mRNA in Ldlr^-/-^ mice with cognitive decline were higher than in C57BL/6 J mice (*t* = 9.79, *P* = 0.009; *t* = 4.68, *P* = 0.009, respectively) whereas the mean levels of Bcl-2 were lower in Ldlr^-/-^ mice than in C57BL/6 J mice (*t* = -7.03, *P* = 0.002; *t* = -8.67, *P* = 0.001, respectively). Moreover, there was no significant difference in caspase-3 mRNA expression (Figure 6D) between the two groups, but activated-caspase3 protein level (Figure 6C) was higher in the Ldlr^-/-^ mice compared with C57BL/6 J mice (*t* = 3.07, *P* = 0.04).

## Discussion

The LDLR is well known for its role in peripheral cholesterol homeostasis [36]; however, it has only been within the last decade has the investigation of cholesterol metabolism in the brain gained momentum [37]. This increased interest is most likely because cerebral cholesterol is implicated in neurodegenerative disease. Here, we used Ldlr^-/-^ mice to imitate the deregulation of cerebral cholesterol. The most striking findings of this study are that Ldlr^-/-^ mice show impaired spatial cognition together with increased intracellular lipid deposition, decreased synapse density and increased neuronal apoptosis in the hippocampus.

Consistent with previous studies [28, 38], our data showed that plasma TC and LDL-c levels in 12-month-old Ldlr^-/-^ mice increased two- and seven-fold, respectively, when fed a standard laboratory chow diet. Unlike humans, LDLR deficiency in mice did not increase plasma lipids significantly. However, ultrastructural studies revealed a rich deposition of intracellular lipid in glial cells, providing direct evidence of the disordered cholesterol metabolism in brains of Ldlr^-/-^ mice. ApoE is generated exclusively by glial cells, and these cells are most likely the source of the cholesterol-apoE particles. The deposition of lipid particles results from a decrease in cholesterol-apoE particle uptake by neurons. Thus, we infer that LDLR deficiency in mice affects cholesterol metabolism in brain rather than the peripheral circulation.

Absence of Ldlr might have (or most likely) contributed to impaired spatial cognition, which was observed previously using the MWM test in 6-month-old mice [39]. They display a decreased number of synaptophysin-immunoreactive presynaptic boutons (SIBPs) in the hippocampus CA1. The MWM test has been proven correlate with hippocampal synaptic plasticity [40]. Our behavioural data were supported by the observation that Ldlr^-/-^ mice displayed a decrease in synapse density/number, as shown by the reduced SIBPs in hippocampal DG other than CA1 regions and the decreased expression of synaptophysin. Ultrastructural examination of Ldlr^-/-^ mice revealed multiple synaptic changes, including decreased vesicles and widened clefts in the DG. A decrease in synapses is one of the characteristics of neurodegenerative diseases [41]. Whether it occurs before the death of neurons remains inconclusive; however, it certainly correlates with neuronal degeneration and death. The hippocampal DG region is one of few brain structures currently known to have high rates of neurogenesis in adult rats [42], contributing to the formation of new episodic memories [43], the spontaneous exploration of novel environments, and other functions [44]. Neurons created in the DG must be established synaptically into the circuitry before they are mature [45]. Synapses contain large membranes in postsynaptic spines and in presynaptic vesicles, which have a particularly high content of cholesterol [46]. A continuous supply of cholesterol is required for synaptogenesis, the results may be explained by the possibility that reduced cellular uptake of cholesterol in Ldlr^-/-^ mice underlies the reduction in synaptic plasticity. In contrast, although the mean cerebral total cholesterol content in Ldlr^-/-^ mice did not differ from that of C57BL/6 J mice [25], research has shown that cholesterol distribution in the plasma membrane is uneven and that the LDLR plays a role in modulating the transbilayer or asymmetric distribution of cholesterol in the exofacial and cytofacial leaflets of the synaptic plasma membranes. Ldlr^-/-^ mice have been shown to display an increase in the percentage distribution of cholesterol and decreased fluidity in the exofacial leaflet [27], which may affect synaptic transmission. Changes intransbilayer or asymmetric distribution of cholesterol may increase Aβ deposition in the brain [47, 48] and enhance synaptic excitotoxicity [49, 50].

The most important feature of AD pathology is neurodegeneration. It has been well documented that some pathological neuronal loss in AD occurs via apoptosis. In the present study, the pro-apoptotic gene bax was expressed at higher levels, and expression of the anti-apoptotic gene bcl-2 was lower at both the mRNA and protein levels in the hippocampus of Ldlr^-/-^ mice. These results indicate that Ldlr^-/-^-mediated distribution of brain cholesterol metabolism led to an increase in apoptosis. Ldlr^-/-^-mediated reduced cellular uptake of cholesterol may promote neuronal death. A deterioration intransbilayer or asymmetric distribution of cholesterol contributes to Aβ deposition, and Aβ oligomers have been shown to induce neuronal apoptosis via the SM-ceramide pathway [51]. In hippocampal DG, Ldlr^-/-^ -induced apoptotic injury potentially suppress the formation of new neurons, and their integration into the neuronal circuitry of the hippocampal network was further hindered through weakening synaptic connections.

Certain limitations existed when we interpreted the data presented in this study. Cholesterol metabolism in mice differs substantially from that in humans. Although it is unknown whether differences in lipoprotein metabolism in the brain exist between mice and humans, a recent cohort study revealed that Ldlr^-/-^ patients showed a six-fold higher incidence of mild cognitive impairment compared with those without Ldlr mutations [22], indicating that LDLR may play similar roles in brain cholesterol metabolism and in maintaining brain function in both humans and mice. Although some ultra-structural changes were observed using transmission electron microscopy, these changes could not be the direct evidence that indicating deterioration in cholesterol homeostasis in the brain. We cannot explain the exact mechanisms that link apoptosis and synapses deficit to the possible brain cholesterol dyshomeostasis. We also should have studied the levels of various neurotransmitters in various areas of the brain, to show that the impaired spatial cognition is only due to synaptophysin but not due to changes in neurotransmitters. But the neurotransmitters in the brain are very complex, the changes of synaptophysin itself may be caused by other neurotransmitters, and vice versa [52–54]. So that it is very difficult to eliminate the effects of these confounding factors. Additionally, the number of animals used for the cellular and molecular biology assays is low. Apoptotic markers like Annexin V or others may be used to quantify the amount of apoptosis.

This study has demonstrated the adverse effects of cholesterol deregulation in the brain on spatial cognition in the Ldlr^-/-^ mouse model. Our findings indicate that LDLR deficiency results in impaired spatial cognition most likely via its deteriorating effect on homeostasis of cerebral cholesterol and negative effects on hippocampal vulnerability to apoptosis and number of synapses. These results will be helpful in understanding the role of LDLR in cholesterol-related cognitive impairment.
